# Assessment of local sensitivity in incomplete retinal pigment epithelium and outer retinal atrophy (iRORA) lesions in intermediate age-related macular degeneration (iAMD)

**DOI:** 10.1136/bmjophth-2024-001638

**Published:** 2024-07-09

**Authors:** Julius Ameln, Marlene Saßmannshausen, Leon von der Emde, Alessandra Carmichael-Martins, Frank G Holz, Thomas Ach, Wolf M Harmening

**Affiliations:** Department of Ophthalmology, University Hospital Bonn, Bonn, Germany

**Keywords:** Imaging, Retina

## Abstract

**Objective:**

Lesions of incomplete retinal pigment epithelium and outer retinal atrophy (iRORA) are associated with disease progression in intermediate age-related macular degeneration (AMD). However, the corresponding functional impact of these precursor lesions is unknown and needs to be established.

**Methods and analysis:**

We present a cross-sectional study of four patients with intermediate AMD employing clinical-grade MAIA (stimulus size: 0.43°, ~125 µm) and adaptive optics scanning light ophthalmoscope (AOSLO, stimulus size 0.07°, ~20 µm) based microperimetry (MP) to assess the specific impact of iRORA lesions on retinal sensitivity.

**Results:**

AOSLO imaging showed overall reduced photoreceptor reflectivity and patches of hyporeflective regions at drusen with interspersed hyperreflective foci in iRORA regions. MAIA-MP yielded an average retinal sensitivity loss of -7.3 ±3.1 dB at iRORA lesions compared to the in-eye control. With AOSLO-MP, the corresponding sensitivity loss was -20.1 ±4.8 dB.

**Conclusion:**

We demonstrated that iRORA lesions are associated with a severe impairment in retinal sensitivity. Larger cohort studies will be necessary to validate our findings.

**WHAT IS ALREADY KNOWN ON THIS TOPIC**

- Incomplete retinal pigment epithelium and outer retinal atrophy (iRORA) lesions are age-related macular degeneration progression markers defined by structural features observed via optical coherence tomography.
- The functional relevance of iRORA lesions is unknown.

**WHAT THIS STUDY ADDS**

- This study established a direct connection between iRORA lesions and localized but severe loss of retinal sensitivity.

**HOW THIS STUDY MIGHT AFFECT RESEARCH, PRACTICE OR POLICY**

- Our findings are further evidence that iRORA lesions have the potential to serve as clinical endpoints for AMD treatment trials at earlier disease stages.

## Introduction

There is an unmet need for prognostic biomarkers and clinical endpoints, approved by regulatory authorities, in early and intermediate age-related macular degeneration (iAMD).^1^ In recent years, high-resolution multimodal retinal imaging, including spectral domain optical coherence tomography (SD-OCT), has identified precursor lesions of geographic atrophy (GA) in AMD.^2^ These have been further characterised by the International Consensus Atrophy Meeting group and termed as incomplete retinal pigment epithelium and outer retinal atrophy (iRORA) in SD-OCT imaging, preceding its later stage, complete retinal pigment epithelium and outer retinal atrophy, cRORA.^3^ While iRORA is highly associated with disease progression,^4^ detailed knowledge of its impact on retinal function is very limited, yet essential for its validation as a potential clinical outcome measure. Conventional functional tests such as clinical-grade microperimetry (MP) are inadequate due to the discrepancy between available test stimuli sizes and the tiny iRORA lesion sizes.

Today, adaptive optics scanning light ophthalmoscope (AOSLO) imaging allows cell resolved visualisation of photoreceptors by correcting the human eyes natural aberrations.^5^ AOSLO systems have been further adapted to allow AOSLO-MP, using the high resolution of the system to facilitate functional sensitivity testing of small retinal areas down to the size of individual photoreceptors.^6–8^ AOSLO-MP has helped to investigate the functional relevance of retinal phenotypes in various diseases, including macular telangiectasia type 2, choroideremia and retinitis pigmentosa.^9–11^

The purpose of this cross-sectional study is to juxtapose retinal sensitivity, gauged through clinical-grade MP and AOSLO-MP, at retinal locations in the presence and absence of iRORA to determine the lesions’ impact on retinal function.

## Methods

### Patients

For this study, patients with large sub-retinal pigment epithelium (RPE) drusen (≥125 µm) associated with iAMD according to the Beckman classification^12^ and at least one iRORA^3^ lesion (region of choroidal hypertransmission <250 µm, zone of attenuation/disruption of the RPE <250 µm, evidence of overlying photoreceptor degeneration in absence of an RPE tear) were recruited. Exclusion criteria were any history of other retinal diseases, glaucoma, a history of vitreoretinal surgery, relevant anterior segment disease with media opacity, cRORA or a history of current or previous anti-vascular endothelial growth factor (VEGF) treatment. If both eyes of a study patient had iRORA, the eye with a more extrafoveal position of the lesion as well as better best-corrected visual acuity (BCVA) was chosen as study eye. All patients underwent routine ophthalmological examination including BCVA, slit lamp and funduscopic examination. Following pupil dilation (0.5% tropicamide, 2.5% phenylephrine), a standardised retinal imaging protocol was performed using SD-OCT raster scanning (241 B-scans, 30°×25° enhanced depth imaging mode, centred on the fovea, lateral scan distance 30 µm, automatic real-time mode 9 frames; Spectralis, Heidelberg Engineering, Heidelberg, Germany). Throughout this study, a retinal magnification factor of 291 µm/deg of visual angle was assumed.

### iRORA grading

**Figure 1:**
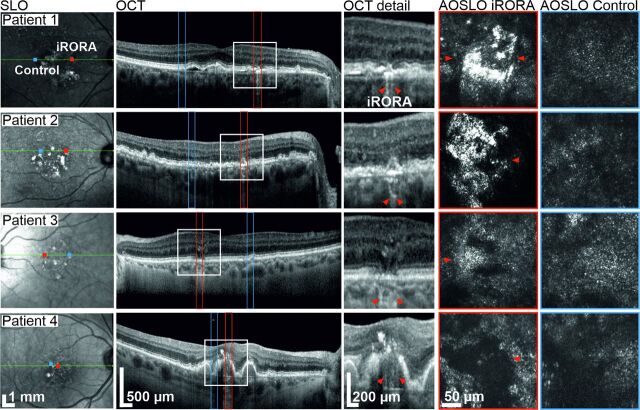
Multimodal imaging of four iAMD patients presenting iRORA lesions. Column 1: Infrared SLO images. OCT B-scan and AOSLO imaging locations are marked (red=iRORA, blue=eccentricity-matched control region). Column 2: OCT-B scan through the centre of the iRORA lesion. Column 3: Magnification of the iRORA lesions marked by the white rectangle in column 2. Red arrow heads mark the extent of the lesion as graded from the OCT scan. Columns 4 and 5 show AOSLO images of iRORA lesions and control regions, respectively. Arrow heads indicate the corresponding locations after OCT and AOSLO image registration. In three cases, only a single marker is visible due to the lesion extending beyond the limited field of view of the AOSLO. AOSLO, adaptive optics scanning light ophthalmoscope; iAMD, intermediate age-related macular degeneration; iRORA, incomplete retinal pigment epithelium and outer retinal atrophy; OCT, optical coherence tomography.

For precise localisation of iRORA, each B-scan of the 241 vol SD-OCT scan was screened by two medical expert graders (MS and LvdE) with specific expertise in iAMD trials. The largest horizontal diameter of the lesion was annotated within the B-scan. For MP testing, in-eye control regions were chosen by eccentricity-matching via mirroring along the foveal vertical meridian. Control regions were allowed to present iAMD-typical structural alterations but required to not contain any iRORA lesions or large blood vessels. In case the requirements were not met, the control region was shifted horizontally or vertically to a valid retinal region, which was found in all cases by shifting less than 100 µm from the initially targeted area. The targeted area was generally smaller than 250 µm edge length, centred on the lesion crossing central B-scan. Per eye, a single iRORA lesion and a single in-eye control region were examined with clinical-grade (MAIA) and AOSLO-MP. Fundus images showing the MP target locations and AOSLO images of the respective regions are shown in figure 1.

### MAIA-microperimetry

For clinical-grade retinal sensitivity testing, the S-MAIA device (CenterVue/iCare, Padova, Italy) was employed (settings: 85 stimuli covering 12° of the central retina, 4–2 dB staircase strategy, stimulus size: 0.43° (Goldmann III)). An initial training session was performed in all patients prior to the main test using achromatic stimuli (400–800 nm). The test duration was about 8–10 min. The background luminance was 4 apostilb (asb or 1.3 cd/m^2^) with a dynamic testing range of 36 dB. The grid stimulation pattern was aligned according to vessel bifurcations to the en-face IR of the SD-OCT image using Fiji, Image J (US National Institutes of Health). Under detailed consideration of the corresponding OCT B-scans, the stimuli points at the position of the respective iRORA lesion were identified.

### Adaptive optics scanning light ophthalmoscope-microperimetry

A custom built dual-channel confocal AOSLO was used to simultaneously image the retina (wavelength 840 nm; field of view: 0.85°) and to deliver visual stimuli at 543 nm (test spot diameter: 0.07°).^7^ The custom AOSLO-MP instrument has been described previously in detail.^7^ In summary, the setup comprised a broadband laser source (SuperK EXTREME; NKT Photonics, Birkerod, Denmark) that was used to provide multiple light channels. The integrated adaptive optics consisted of a Shack-Hartmann wavefront sensor (SHSCam AR-S-150-GE; Optocraft, Erlangen, Germany) and a deformable mirror (DM97-08; ALPAO, Montbonnot-Saint-Martin, France) in closed loop. Wavefront sensing was performed using the imaging wavelength. Two acousto-optic modulators (TEM-250-50-10-2FP; Brimrose, Sparks Glencoe, Maryland, USA) in cascaded configuration allowed generation of high-contrast visual stimuli at a wavelength of 543 nm.^7 8^ The 840 nm AOSLO imaging field created a constant background illumination of 13.2 asb (4.2 cd/m^2^) against which the 543 nm test spots were shown. Visual stimuli could be presented over a 50 dB dynamic range.^7 8^

The position of the patient’s head was controlled using a dental impression stage (bite bar). Patients were instructed to fixate on a small visual annulus presented via a pellicle beam splitter during imaging and testing. A 4–2 dB descending staircase strategy with three threshold crossings was used. Sensitivity thresholds at both iRORA and control test sites were defined in dB as the average from at least five valid repeat runs per location (five at iRORA and five at control region). Runs where catch and lapse trials were notable, or where the patient or the supervisor noted down any issue, were excluded from the analyses. Prior to testing, AOSLO images were recorded at the relevant retinal locations. Patients then performed several practice runs to familiarise themselves with the test procedure. While each single AOSLO-MP test run took less than a minute to complete, the total time for AOSLO imaging and AOSLO-MP took about 1 hour per eye.

### Statistical analysis

A one-sided two-sample t-test was carried out using the ttest2 Matlab function to test for statistical significance (p<0.05) of AOSLO-MP sensitivity loss in the presence and absence of iRORA.

## Results

**Figure 2:**
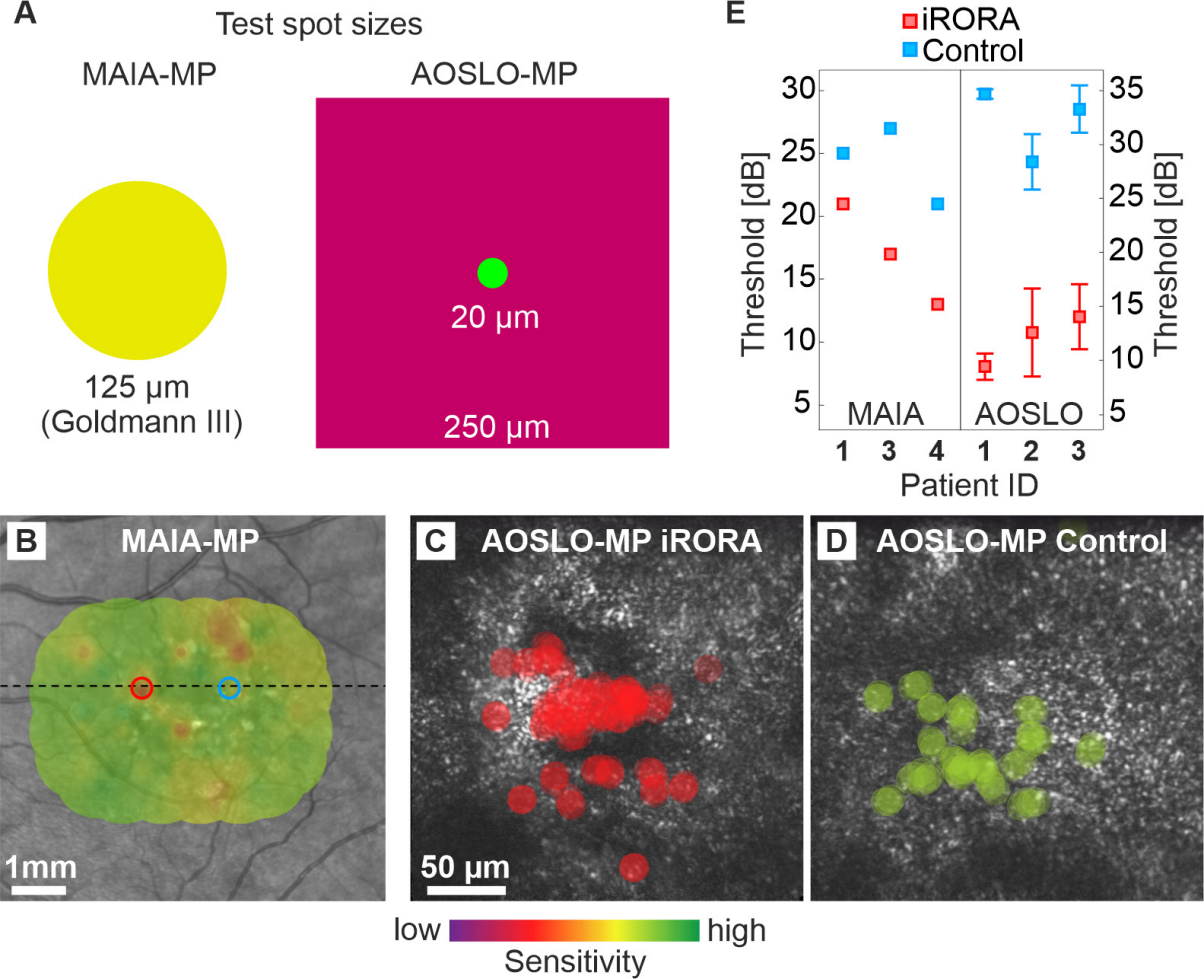
Retinal sensitivity at iRORA lesions and control regions in iAMD. (A) Test spot size comparison between MAIA-MP (achromatic stimulus) and AOSLO-MP (543 nm stimulus within a larger 840 nm raster). (B) MAIA-MP estimate of retinal sensitivity at the iRORA lesion (17 dB, red) and control region (27 dB, blue) in patient #3. The marker diameter corresponds to MAIA-MP stimulus size on the retina. (C, D) AOSLO-MP test spot size and location at the iRORA lesion (C) and control region (D) in patient #3. Sensitivity thresholds were 14 dB and 33.3 dB, respectively. Both areas show patches of normal hexagonally arranged cone photoreceptors and areas of hyporeflective retina. (E) Sensitivity thresholds at the iRORA lesions and control regions for MAIA-MP (patients #1, 3, 4) and AOSLO-MP (patients #1, 2, 3). AOSLO, adaptive optics scanning light ophthalmoscope; iRORA, incomplete retinal pigment epithelium and outer retinal atrophy; MP, microperimetry.

Four eyes of four iAMD patients (mean age: 73, range: 60–85) were included. Detailed patient characteristics are given in table 1. Multimodal imaging of iRORA and control regions is shown in figure 1. When averaged across all four test eyes, the loss of sensitivity at the iRORA site relative to the control site was 20.1±4.8 dB for AOSLO-MP and 7.3±3.1 dB for MAIA-MP (figure 2).

**Table 1:** Patient ocular characteristics and retinal sensitivity thresholds

					MAIA-MP threshold (dB)		AOSLO-MP threshold (dB)	
Patient	Sex	Eye	Lens status	BCVA (logMAR)	iRORA	Control	iRORA	Control
# 1	F	OD	Phakic	−0.1	21	25	9.4±1.2	34.7±0.4
# 2	M	OD	Phakic	0.1	–	–	12.6±4.1	28.4±2.6
# 3	F	OS	Phakic	0.0	17	27	14.0±3.0	33.3±2.2
# 4	F	OD	Pseudophakic	0.4	13	21	–	–

If available, averages±1 SD are reported.

MAIA-MP was not available in patient #3, AOSLO-MP was not available in patient #4.

AOSLO, adaptive optics scanning light ophthalmoscope; BCVA, best-corrected visual acuity; iRORA, incomplete retinal pigment epithelium and outer retinal atrophy; logMAR, logarithm of the minimum angle of resolution; MP, microperimetry.

### Patient presentation

Patient #1 presented with hyper-reflective material and hyporeflective patches at the photoreceptor level at the iRORA lesion, surrounded by structurally altered photoreceptors. The control region showed an irregular photoreceptor mosaic with large patches of slightly hyporeflective photoreceptors (figure 1). AOSLO-MP revealed a retinal sensitivity threshold of 9.4 dB at the iRORA lesion and of 34.7 dB (25.3 dB difference, p<0.01 two-sample t-test) at the control region (figure 2). The corresponding MAIA-MP sensitivity difference was 4 dB.

Patient #2 iRORA lesion and control region displayed morphologies similar to Patient #1. AOSLO-MP was successful, showing a significant retinal sensitivity loss between iRORA lesion and control region (15.8 dB, p<0.01) while MAIA-MP failed due to patient fatigue (table 1, figure 2).

Patient #3 displayed patches of healthy-appearing photoreceptors surrounded by less reflective and irregularly arranged photoreceptors, as well as regions of hyporeflective photoreceptors, at the location of the iRORA lesion. The respective control region had a similar appearance (figure 1). AOSLO-MP revealed a sensitivity loss of 19.3 dB between iRORA and control (p<0.01), the MAIA-MP sensitivity loss was 10 dB (figure 2).

Patient #4 iRORA lesion appeared as a central black area surrounded by remnant enlarged cone structures. AOSLO-MP failed due to insufficient fixation stability of the patient. MAIA-MP showed a significant retinal sensitivity loss between the iRORA lesion and control region (8 dB, p<0.01) (table 1, figure 2).

## Discussion

We here report the immediate impact of iRORA lesions on retinal function in four patients with iAMD. iRORA is a structural precursor for GA development but can now also be linked to functional impairments detected by AOSLO-MP.

The image signal in confocal AOSLO stems from the reflective properties of the photoreceptors’ outer segments. Patches of less reflective and irregularly arranged photoreceptors, as seen in our patients, are thus highly suspicious of functional and structural impairment. Specifically, the displacement of the cells, that is, by drusen, as well damage to the outer segments are likely causes of the observed changes. The photoreceptor mosaic phenotype at iRORA lesions here reported (see figure 1), seems to be comparable to previously reported structural changes at retinal locations with iAMD drusen (patient #3) or GA (patient #4).^13 14^ Interestingly, while all iRORA lesions showed abnormalities in photoreceptor arrangement and reflectivity, the impact on function differed across patients. It is hypothesised that with iRORA progression, photoreceptors will further deteriorate. However, photoreceptor loss in areas of iRORA cannot be readily quantified and compared with control regions by confocal AOSLO, given the impact of photoreceptor outer segment health changes on this imaging modality.

In all our patients, a significant loss of retinal sensitivity at the iRORA lesions was detectable. While both MAIA-MP and AOSLO-MP results demonstrate functional loss, its magnitude is about four times higher in AOSLO-MP, on average. We suggest that this difference is explained by the smaller and better targeted stimulus available for AOSLO-MP. The ~6 times larger Goldmann 3 stimulus used for MAIA-MP likely includes areas not affected by the small iRORA lesions and elicits retinal responses by comparably healthy adjacent photoreceptors (compare patient #1 and #3). The steep retinal sensitivity loss detected with AOSLO-MP indicates that iRORA lesions can be considered as a surrogate marker for retinal dysfunction. A limitation of our study is the fundamentally different setup of the MAIA-MP and AOSLO-MP. The AOSLO-MP background illumination is about 3.3 times brighter due to light requirements of the adaptive optics components used for wavefront correction and retinal imaging. Furthermore, the MAIA-MP uses white stimuli, while green (543 nm centre wavelength) stimuli are used for AOSLO-MP, selected for equal sensitivity in the long-wavelength and middle-wavelength sensitive cones. Additionally, we have a small shared patient pool. One patient failed the MAIA-MP (patient fatigue), and one failed the ASLO-MP (insufficient fixation stability), resulting in only two patients with shared results for both MAIA-MP and AOSLO-MP out of a total of four recruited patients.

## Conclusions

This pilot study revealed a statistically significant and severe decrease in retinal sensitivity at iRORA lesions in iAMD patients. Larger cohort studies will be necessary to validate our findings.
